# Antiviral activities and applications of ribosomally synthesized and post-translationally modified peptides (RiPPs)

**DOI:** 10.1007/s00018-021-03759-0

**Published:** 2021-02-02

**Authors:** Yuxin Fu, Ate H. Jaarsma, Oscar P. Kuipers

**Affiliations:** 1 Department of Molecular Genetics, Groningen Biomolecular Sciences and Biotechnology Institute, University of Groningen, 9747 AG Groningen, The Netherlands; 2 Department of Environmental Science, Aarhus University, 4000 Roskilde, Denmark

**Keywords:** RiPPs, Antiviral activity, Antiviral mechanism, Engineering

## Abstract

The emergence and re-emergence of viral epidemics and the risks of antiviral drug resistance are a serious threat to global public health. New options to supplement or replace currently used drugs for antiviral therapy are urgently needed. The research in the field of ribosomally synthesized and post-translationally modified peptides (RiPPs) has been booming in the last few decades, in particular in view of their strong antimicrobial activities and high stability. The RiPPs with antiviral activity, especially those against enveloped viruses, are now also gaining more interest. RiPPs have a number of advantages over small molecule drugs in terms of specificity and affinity for targets, and over protein-based drugs in terms of cellular penetrability, stability and size. Moreover, the great engineering potential of RiPPs provides an efficient way to optimize them as potent antiviral drugs candidates. These intrinsic advantages underscore the good therapeutic prospects of RiPPs in viral treatment. With the aim to highlight the underrated antiviral potential of RiPPs and explore their development as antiviral drugs, we review the current literature describing the antiviral activities and mechanisms of action of RiPPs, discussing the ongoing efforts to improve their antiviral potential and demonstrate their suitability as antiviral therapeutics. We propose that antiviral RiPPs may overcome the limits of peptide-based antiviral therapy, providing an innovative option for the treatment of viral disease.

## Introduction

### The viral threat to human health

Viruses represent a major cause of disease and mortality worldwide. Although great progress has been made in virus control, approved antiviral therapies for the majority of viruses are still lacking [1–4]. Many of these viruses are enveloped. The envelope is comprised of a host-derived membrane and surrounds the capsid, which is the protein shell that protects the viral genome [5]. In recent times, several epidemics have been caused by emerging new enveloped viruses, such as Human Immunodeficiency Virus (HIV), influenza, Zika, Ebola and Coronaviruses like severe acute respiratory syndrome (SARS-CoV), Middle East respiratory syndrome (MERS-CoV), and SARS-CoV-2 [6–8]. It is evident that emerging viruses, for which there is no therapy developed yet, are a big threat. Furthermore, effective drugs can be rendered useless due to rapidly mutating viruses, leading to viral drug resistance [9]. These factors illustrate that control over viral disease is still a major challenge. They trigger, next to vaccines, the development of novel antiviral therapeutics for human disease.

### Current strategies in antiviral therapy

Apart from vaccines, various antiviral drugs exist that inhibit the course of viral infections in humans. One strategy currently used, is the targeting of host proteins and pathways that are essential for viral infection. Broad spectrum antivirals can potentially be developed by targeting host pathways that are exploited by multiple viruses [10]. However, analysis of approved antiviral drugs shows that a majority of these drugs are virus-targeting rather than host-targeting [11], indicating that this type of broad-spectrum antivirals is still underrepresented. Direct acting antivirals can inhibit a virus directly, by acting on the different steps in the viral infection pathway. For example, the first steps of invasion that can be blocked are the attachment to and subsequent entry into the host cell. Other antiviral agents target the key processes that are required for virus proliferation in the host cell, by inhibiting the essential enzymes of the virus. Protease inhibitors can, for instance, block the release of functional viral proteins from a polyprotein precursor. Similarly, polymerases provide a good target for antivirals, as most viruses require polymerases for replication and transcription. Examples of such drugs are nucleoside and nucleotide analogs, and allosteric site inhibitors. Other viral enzymes that can serve as targets for antivirals are integrases (in retroviruses), methyltransferases, or helicases [12].

Although effective antivirals have been developed, sufficient therapies to fight the majority of viruses that cause human disease are still lacking. Notably, there is also an asymmetry in the type of infections for which drugs are approved. A majority of approved drugs target chronic infections such as those caused by HIV and hepatitis causing viruses, rather than acute viral infections, like influenza [11]. In addition, a narrow spectrum of activity, and emergence of resistance are serious limitations of the existing direct-acting antivirals with a very specific target [10]. In combination with the prevalence of chronic viral diseases and the (re-)emergence of new viruses, for which no vaccines exist yet, these problems showcase the clear need for novel antivirals, especially those with a broad activity spectrum [12]. There is an increasing number of studies demonstrating the antiviral activity of peptides, including some antimicrobial peptides and innate host defense peptides, the discovery, activity and therapeutic potential of such molecules have been previously reviewed [13, 14]. Peptides have a larger interaction area and, therefore, greater potential for forming specific and high-affinity interactions with targets than small molecules. Moreover, it has been demonstrated that (modified) peptides can target large protein–protein interaction sites that are too large to be accommodated by small antiviral molecules [15–17]. RiPPs are highly modified peptides (see next section) giving rise to numerous different epitopes and, thus, provide high structural diversity. These advantages further support the development of peptide-based antivirals. However, some potential drawbacks of linear peptides such as short half-life, immunogenic potential, high cost of production, and poor oral bioavailability, still limit the use of linear peptides in pharmaceutical industry [13].

### Antiviral potential of RiPPs

Ribosomally synthesized and post-translationally modified peptides are a class of natural products, produced by all three kingdoms of life. Extensive enzymatic post-translational modifications of these peptides yield chemically exotic structures [18, 19]. Often, conformational flexibility is constrained by these modifications, to improve for instance stability or target recognition. As a result, RiPPs commonly have increased protease resistance or high specificity as a benefit over unmodified peptides. Various RiPPs have a strong antibacterial activity, and as such are extensively studied as novel antibiotics. Many of these antimicrobially active RiPPs target the bacterial membrane. The fact that RiPPs are gene encoded, and that their biosynthetic pathways can be recombined, greatly benefits the engineering of novel structures. Through mutagenesis and by shuffling modification enzymes from different biosynthetic gene clusters, novel RiPPs can be designed, with, for example, stronger antimicrobial potency, better stability, or a broader activity spectrum [18, 20, 21].

**Fig. 1:**
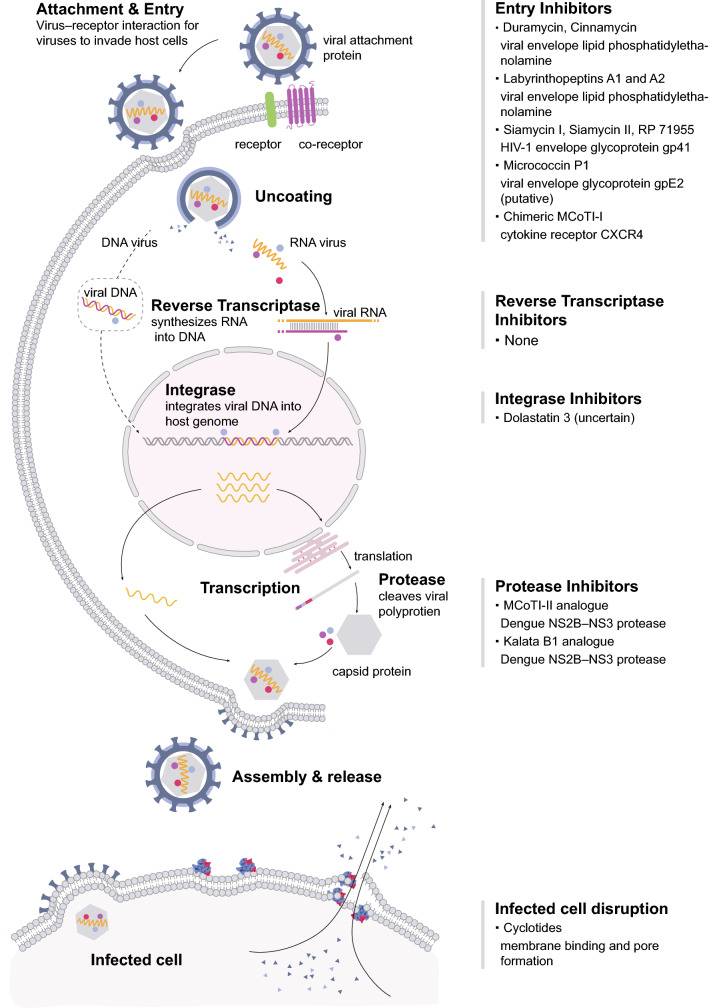
Schematic model of known antiviral mechanisms of RiPPs. Antiviral RiPPs exert their activity at different phases of the viral infection process. In the column on the right, several antiviral RiPPs are grouped based on their antiviral phases and mode of action. If known, the specific target is also shown

While RiPPs are being well studied in the context of antibacterial activity, various RiPPs that exhibit antiviral activity also exist in nature. Some of them that target lipid membranes are of interest for application against broad spectrum enveloped viruses, which form a particularly dangerous class of viruses. Thus, their structures may provide promising leads for the engineering of novel antivirals. We note that the antiviral potential of RiPPs in treatment of viral disease may have been largely overlooked. In this review, we explore the antiviral RiPPs that exist in nature, their mechanism of action (Fig. 1), the possibilities for the engineering of them, and discuss their potential for therapeutic application.

### Natural antiviral RiPPs

**Fig. 2:**
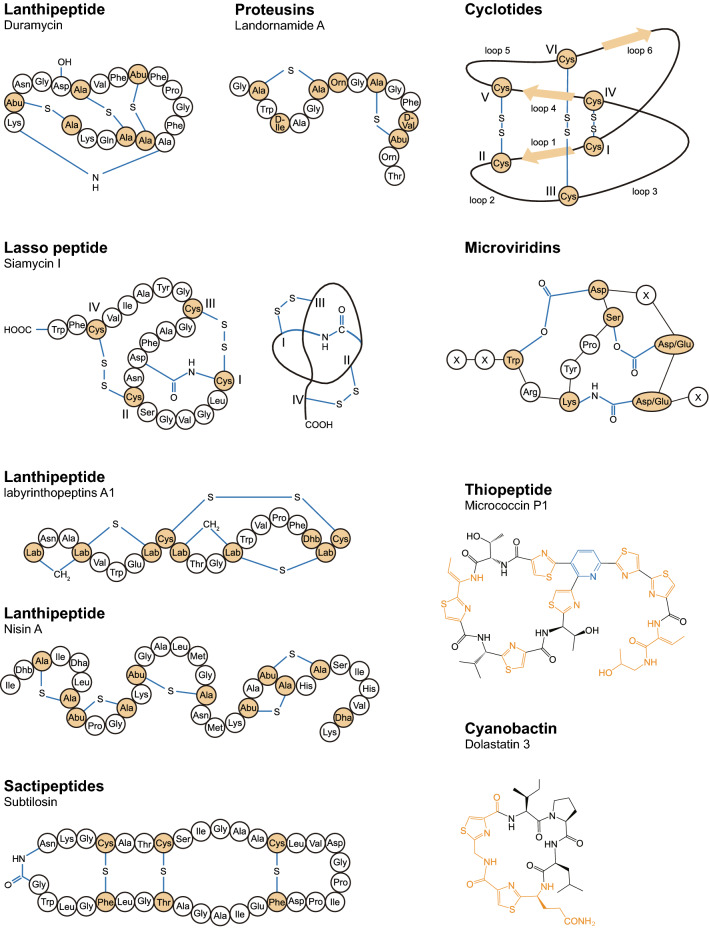
Representative structures of eight selected RiPP families. Post-translational modification(s) on each structure are highlighted in yellow and blue. *Abu* α-aminobutyric acid, *Dha* dehydroalanine, *Dhb* dehydrobutyrine, *Orn* Ornithine, *lab* labionin

Ribosomally synthesized and post-translationally modified peptides are an ever-expanding group of peptidic natural products with diverse chemical structures and biological activities [18]. Examples of some RiPPs structures, some with antiviral activity, are depicted in Fig. 2, illustrating their structural diversity. They have emerged as a major category of secondary metabolites partly due to the use of efficient genome mining methods [22] that have led to a myriad of microbial genome sequencing and biosynthetic studies in the past two decades. Recent evidence highlights the function of antiviral peptides as a defensive barrier. It has been demonstrated that some RiPPs present activity against a broad range of viruses, hereafter referred to as antiviral RiPPs (Table 1). The extensive post-translational modification of RiPPs endows them with excellent stability, diverse three-dimensional structures, and biological properties such as lipid-binding activity and membrane permeability. These important biopharmaceutical characteristics, as well as their relatively small size (2–5 kDa), make them good candidates for novel drug development.

**Table 1:** Collection of reported antiviral RiPPs

RiPPs class	Peptides	Targeted virus	Mechanism of action
	Nisin	BVDV	Interact with the negatively charged viral capsid (hypothesis)
	Duramycin	Ebola/DENV/WNV/ZIKV	Prevent viral entry by binding viral envelope lipid PE
Lanthipeptide	Cinnamycin	HSV-1	Prevent viral entry by binding viral envelope lipid PE
	Labyrinthopeptin A1 Labyrinthopeptin A2	HIV/HSV/RSV/DENV/ZIKV/WNV/HCV/CHIKV/KSHV/CMV	Prevent viral entry mainly by binding viral envelope lipid PE; disrupt virus particle membrane integrity
	Divamide A	HIV	
Lasso peptide	RP 71,955	HIV	Prevent viral fusion by interfering with the molecular function of viral glycoprotein gp41 (hypothesis)
	Siamycin I	HIV	Same as above
	Siamycin II	HIV	Same as above
	Specialicin	HIV	
	Humidimycin	HIV	
Cyclotides	Circulin A,B	HIV	
	Cycloviolins A-D	HIV	
	Circulin C-F	HIV	
	vhl-1	HIV	
	Tricyclon A	HIV	Membrane binding and disruptive action
	Cycloviolacin Y1, Y4, Y5	HIV	
	Cycloviolacin VY1	Influenza A (H1N1)	
	Cycloviolacin O2	HIV	Membrane binding and disruptive action
	Cycloviolacin O13	HIV	
	Kalata B1	HIV	Membrane binding and disruptive action
	Varv peptide E	HIV	
	Cycloviolacin O14, O24	HIV	
	Palicourein	HIV	
	Kalata B8	HIV	Membrane binding and disruptive action
Proteusins	Landornamide A	LCMV	
Bacterial head-to-tail cyclized peptides	Enterocin B	Influenza viruses	
Thiopeptides	Micrococcin P1	HCV	Prevent viral entry by interfering with viral glycoprotein E2 (putative)
Sactipeptides	Subtilosin	HSV-1	
Cyanobactin	Dolastatin 3	HIV	Inhibit HIV integrase (uncertain)
Conopeptides	Asteropine A		

*HIV* human immunodeficiency virus, *HSV* herpes simplex virus, *HSV-1* herpes simplex virus type 1, *RSV* respiratory syncytial virus, *DENV* dengue virus, *ZIKV* zika virus, *WNV* west nile virus, *HCV* hepatitis C virus, *CHIKV* chikungunya virus, *KSHV* kaposi’s sarcoma-associated herpesvirus, *CMV* cytomegalovirus, *Ebola* ebola virus *BVDV* bovine viral diarrhoea virus, *Influenza A(H1N1)* influenza A virus subtype H1N1

### Antiviral lanthipeptides

Lanthipeptides are defined by the presence of the characteristic thioether cross-linked amino acids lanthionine (Lan) and/or methyllanthionine (MeLan), and represent the largest class of RiPPs. The lanthionine-containing peptides that have antimicrobial activity are called lantibiotics, but recent research has revealed that lanthipeptides can have other functions beyond antimicrobial activities, such as antiviral activity, on which we are focusing in this review.

Lanthipeptides are classified into four different classes depending on the biosynthetic enzymes that install the Lan and MeLan motifs. Nisin is a representative of Class I lanthipeptides. With an overall positive charge and amphipathic properties, nisin shows broad-spectrum antimicrobial activity against Gram-positive bacteria, while recent studies suggest that these properties can also facilitate nisin to interact with the negatively charged viral capsid and verified its influence on Bovine Viral Diarrhea Virus (BVDV), an enveloped RNA virus [23, 24]. The 50% effective concentration (EC_50_) of nisin against BVDV is around 12 μg/ml, which is lower than its 50% cytotoxic concentration (CC_50_) of 167 μg/ml. A mode of action study stated that the inhibitory effect of nisin was present throughout the entire duration of viral infection (adsorption and post-adsorption), and the intensity of the inhibitory effect occurred in a dose-dependent manner and did not weaken with the increasing incubation times. Further research revealed that nisin does not have direct virucidal activity, i.e., no significant influence was found on the titre of the virus after direct contact with virus particles [23]. Therefore, determining how nisin exerts its antiviral effect and whether it exerts an indirect antiviral effect through electrostatic interaction or other actions still needs in-depth research. Although the precise antiviral mechanism of nisin is unclear, it has been proven to have an effective antiviral effect in combination with other antiviral compounds [23], which shows that nisin is still expected to be developed as a viral inhibitor to supplement other antivirals to protect human or animal cells.

Duramycin and Cinnamycin are examples of class II lanthipeptide with antiviral activity [25, 26]. They are 19-amino acid cyclic peptides with only one amino acid is different in position 2. Besides Lan and MeLan motifs, they have an unusual lysinoalanine (Lal) bridge linked by lysine 19 and serine 6, and an β-hydroxylated aspartic acid at position 15 (Fig. 2); these modifications are important to reach unusually high affinity (with a dissociation constant of 4–6 nM) and specificity of peptides with their antiviral target, phosphatidylethanolamine (PE). The previous study indicated that the Lal linkage is critical to set up the PE-binding pocket and the binding is stabilized by an ionic interaction between β-hydroxylated aspartic acid and the head group of PE, leading to a tight complex which confers peptides with an exclusive specificity for PE [27–31]. PE is present at the surface of enveloped viruses and is not exposed on the surface of healthy cells [32], it is a ligand for phosphatidylserine (PS) receptors and promotes PS-mediated viral entry. Thus, it was suggested to be attractive target for the design of broad-spectrum antiviral therapies [25]. With high PE-binding affinity, Duramycin works at step of virus adsorption and effectively block the entry of various pathogenic viruses, such as Ebola, Dengue, West Nile [25] and Zika viruses [33]. Similarly, cinnamycin has good antiviral activity and inhibited the cytopathic effect of HSV-1 with a 50% inhibitory dose (ID_50_) of 0.05 μg/ml [26]. For viral lipid PE targeting antivirals, they have advantages to be developed with a broader activity spectrum and less chance of resistance development, since PE is widely present in enveloped viruses and plays a central role in viral infection [34–36]; at the same time, lipids are less susceptible to mutability and rapid evolutionary changes than protein or glycan motifs [37]. The reported hemolytic activity of duramycin and cinnamycin seems to hamper their antiviral therapeutic potential [38]. However, the sensitivity of hemolysis differed greatly among animal species [39], thus requiring a more thorough toxicological studies at therapeutic concentrations, and the further optimization could be achieved by RiPPs engineering, as will be discussed in the next section.

In 2018, divamide A, a close homolog of cinnamycin discovered from an extract of marine tunicates, was reported to display potent anti-HIV activity without being notably cytotoxic. The divamides represents a novel family of lanthipeptides. Divamide A was predicted to have a novel lanthipeptide structure, with three MeLan, a Lal, a β-hydroxylated aspartic acid, and a N-terminal trimethylation, a rare post-translational modification [40]. Some divamide-like peptides have the same constrained shape, but differ only in amino acid sequences with divamide A. They lack any apparent anti-HIV effects and show some cytotoxic effect [40]. A structure-activity relationship study on divamide A using a series of analogs showed that Lal is necessary for its anti-HIV activity, because all derivatives containing Lal were active with similar potency. Only derivatives, in which the Lal residue was not present, were inactive. These results imply that subtle changes in the divamide sequence can have significant effects on antiviral and cytotoxic properties. Additionally, the cytotoxicity and anti-HIV activity relationship of divamide A revealed that it has a wide therapeutic index and the prominent anti-HIV activity stems from its effective segregation of antiviral and cytotoxic properties [40]. In fact, marine organisms are a rich source of unprecedented compounds with different bioactivity. Several antivirals have already been identified from marine organisms. Along with the development of approaches for accessing interesting new bioactive peptides in marine cultures and extraction of these peptides, it appears promising to explore the marine environment for more RiPPs with significant antiviral activity.

Besides duramycin and cinnamycin, the class III lanthipeptides labyrinthopeptins A1 and A2 (Laby A1/A2) also show virolytic activity through binding to the viral envelope lipid PE, thereby inhibiting a broad range of viruses. Labyrinthopeptins producing strain *Actinomadura namibiensis* was isolated in 1988, but Labyrinthopeptins structures were only determined recently [41, 42]. Their structures are characterized by a unique carbocyclic post-translationally modified amino acid named labionin (Lab) [42] (Fig. 2). Laby A1 was initially reported to have anti-Herpes simplex virus (HSV) activity comparable to that of the anti-herpetic drugs acyclovir and cidofovir, with EC_50_s ranging between 0.29 and 2.8 µM. Moreover, a strong activity of Laby A1 against broad-spectrum human HIV (EC_50_s range from 0.79 to 3.3 µM) was found in vitro and it was realized that Laby A1 interferes with the HIV entry process, presumably by acting as an adsorption/co-receptor/fusion inhibitor. LabyA2 also showed antiviral activity against HSV, but was at least tenfold less potent than Laby A1 [43]. Recently, a study reported that Laby A1/A2 can inhibit the infection of cells by a series of enveloped viruses, including Dengue, Zika, West Nile, Hepatitis C, Chikungunya and Karposi’s Sarcoma-associated Herpes virus, with 50% inhibitory concentration (IC_50_) in the low micromolar to nanomolar range, and it was clarified that Laby A1/A2 specifically interact with the envelope lipid PE (rather than sphingomyelin with an ethanolamine head group, phosphatidylcholine, or other eight lipid species tested), thereby disrupting the membrane to exert their antiviral effect [37]. Subsequently, the result of another mechanistic study supported the notion that an interaction between Laby A1/A2 and PE is critical for their antiviral activity, and verified that labyrinthopeptins exert virolytic effect on Respiratory syncytial virus (RSV) by disrupting virus particle membrane integrity [44]. Their anti-RSV effect was confirmed in primary human airway cells ex vivo and with an in vivo murine model [44]. In addition, a clear synergetic effect was observed when Laby A1/A2 were applied in combination with each other. It is worth noting that Laby A1/A2 have advantages over duramycin and cinnamycin in low cytotoxicity and lack of adverse drug effects. Thus, labyrinthopeptins are promising antivirals to be developed with efficacy against a broad range of viruses, and with favorable therapeutic index [37, 44].

### Antiviral lasso peptides

Lasso peptides are a class of RiPPs that are characterized by their unique lasso structure [18] (see also Fig. 2). This lasso structure is formed by an isopeptide bond between the N-terminus and the side chain carboxylate of Asp/Glu to form a 7–9-membered ring through which the remaining C-terminal part of the peptide is threaded and locked in place. This constrained structure confers remarkable stability and strong biological activity to them [45].

Lasso peptides are divided into four classes [46]. The first class is characterized by two disulfide bonds and the first residue always being a cysteine [18]. Interestingly, the lasso peptides with antiviral activity reported so far all belong to this class. These are rifamycin I (or NP-06), siamycin II, RP 71,955, specialicin and humidimycin, respectively. The amino acid sequence of these lasso peptides is similar and they only differ in three amino acids at positions, 4, 8 and 17, Structural analysis indicated that they possess a similar conformational structure [47, 48], and it is most likely that they have similar mechanisms of action. Also, there are reports that synthetic linear polypeptides with an identical amino acid sequence as siamycin I or RP 71,955 loose the activity against HIV, further illustrating that the structural rigidity of these peptides plays an important role in their antiviral activity [49, 50].

RP 71,955 was the first lasso peptide reported with antiviral activity, which was in the context of developing novel agents that can perturb other steps of the virus life cycle in addition to inhibition of the HIV-specific enzymes reverse transcriptase and HIV protease [50]. RP 71,955 inhibits HIV replication in cell culture and shows low cytotoxicity. In the process of exploring its mechanism of action, initially, because of its highly hydrophobic character, it was speculated that RP 71,955 likely interacts with membranes for its antiviral action [50]. Then, a 33% sequence identity was observed between RP 71,955 and part of an immunodominant loop region of glycoprotein gp41 [50, 51]. Gp41 and gp120 are cleaved from precursor envelope proteins gp160. Gp41 as a transmembrane protein of HIV-1, which mediates fusion of the virion once surface glycoprotein gp120, has attached the virus to target cells via binding to the CD4 receptor and a co-receptor [52]. Another interesting finding is that the FXG motif usually present in the fusion domains of gp41, can also be found in one of the *β* turns of RP 71,955 (residues 10–12). Thus, it was hypothesized, suggested by these sequence homologies and predicted conformational similarity, that the anti-HIV effect of RP 71,955 was achieved by interfering with the molecular function of gp41 [50]. And there are indeed some reports showing that synthetic peptides derived from the HIV gp41 sequence function as fusion inhibitor against HIV, such as Enfuvirtide (derived from the HIV-1 gp41 CHR domain), N36 (derived from the HIV-1 gp41 NHR domain) and a series of derivative peptides, which have recently been reviewed [53–55]. Soon afterwards, the anti-HIV activities of the lasso peptides siamycin I and siamycin II were revealed, showing that siamycin I can inhibit the replication of a primary HIV-1 isolate with an IC_50_ value of 1.3 μM [49, 56]. Initially, they were proposed to act as inhibitor of fusion between HIV gp160 and CD4-expressing cells [56], but siamycin I affected neither the gp120-CD4 binding nor the cell surface expression of CD4 [57]. In fact, gp41 appears to be the most likely target [47]. Analysis of structural data for siamycins revealed that the peptides are wedge-shaped, with one face being predominantly hydrophobic, and the other predominantly hydrophilic. This amphipathic character of siamycins may determine their target-binding mechanism, thus playing a role in inhibiting HIV-induced cell fusion. Assuming that the hydrophobic face of the siamycins may interact with the hydrophobic gp41 fusion domain, this would interfere with membrane penetration of the fusion domain, or trap gp41 in an inactive conformation [47]. A subsequent study found that siamycin I exerts its activity through a noncovalent interaction [57], further delineating the binding mode of these peptides. Constructing mutant variants of these lasso peptides could be helpful to deeper understand the structure-antiviral activity relationship and what is required for inhibition.

In recent years, two new anti-HIV class I lasso peptides, i.e., specialicin (active against HIV-1 NL4-3 with IC_50_ around 7.2 µM) [48] and humidimycin [58] were identified based on genome mining, then isolated and characterized. The structural elucidation showed that they are closely related to siamycins, possessing anti-HIV activity with no significant toxicity. Given the limited number of class I lasso peptides and their high similarity, it might be expected that there are more class I lasso peptides that inhibit HIV.

### Antiviral cyclotides

Cyclotides are plant-derived RiPPs that are characterized by a head-to-tail cyclic peptide backbone and a signature cyclic cystine knot (CCK) motif arrangement of three conserved disulfide bonds [18] (see also Fig. 2). They represent the first family of gene-encoded cyclic peptides discovered in plants [59]. The unique structure makes them exceptionally resistant to chemicals, heat and proteolysis, which is a useful biopharmaceutical characteristic for therapeutic molecules [60]. Cyclotides exhibit a variety of biological activities. Among them, anti-HIV activity has been one of the most extensively studied so far, due to its potential pharmacological applications. The first cyclotides with anti-HIV activity were reported as an outcome of a HIV-inhibitory natural products screening program [61]. Two cyclized peptides, circulin A and circulin B, were identified and verified to inhibit replication and the cytopathic effect of HIV infection with an EC_50_ of approximately 70 nM [62]. By subsequent sequence examination and structure determination, cyclotides were classified into two main subfamilies, i.e., Möbius and bracelet, based on the presence or absence of a conceptual backbone twist caused by a conserved *cis*-Pro residue in loop 5. Circulin A and circulin B, without this twist, are referred to as bracelet cyclotides [63, 64]. As more and more cyclotides are isolated and identified, it becomes clear that most of them with antiviral activity fall into the class of bracelet cyclotides, including cycloviolins A-D [65], vhl-1 [66], circulins C-F [67], cycloviolacin O13 [68], cycloviolacin Y1, Y4, Y5 [69], tricyclon A [70], cycloviolacin O2 [71] and cycloviolacin VY1, the first cyclotide reported with anti-influenza A H1N1 virus activity with an IC_50_ value around 2.27 μg / mL [72]. Unfortunately, some studies also show that these antiviral bracelet cyclotides, except for cycloviolacin Y1, Y4 and Y5, typically exert cytotoxic effects on the host cells, thus having a low therapeutic index [62, 65, 73]. Since the only antiviral members of the Möbius subfamily, kalata B1 [74], Varv peptide E [75], cycloviolacin O14 and O24 [68] were reported to have a comparable HIV-inhibitory activity to bracelet cyclotides, but with a reduced toxicity to the target cells, they can be regarded as more promising leads in anti-HIV therapy than their bracelet counterparts. The difference in antiviral properties between these two main cyclotides subfamilies led to an exploration of the relationship between structure and antiviral activity. Moreover, cyclotides also contain a smaller, third group, which is referred to as the trypsin inhibitor subfamily. Currently composed of two members, named MCoTI-I and MCoTI-II [61, 76], these two peptides have highly divergent amino acid sequences, and do not show significant sequence homologies to other cyclotides, but the six conserved cysteine residues that form a typical CCK motif are still present. In addition to the three subfamilies mentioned, hybrid cyclotides with anti-HIV activity have already been reported. They appear to be a hybrid of the two major subfamilies (Möbius and bracelet) of cyclotides, such as kalata B8 [77] and palicourein [78]. Kalata B8 displays anti-HIV activity, but has no significant sequence similarity to most of the published cyclotides. This reinforces the conclusion from other studies that sequence variation between the subfamilies does not appear to influence the level of activity, but that the intact CCK fold of cyclotides is essential for their antiviral activity [74].

Although the exact antiviral mechanism of action is not fully understood, previous studies have suggested that the antiviral activity of some cyclotides is dependent on their membrane-binding and disruption properties [79–82]. Besides the conserved CCK motif, hydrophobic and electrostatic characteristics of these peptides are important for membrane binding as well [59, 69, 83]. In addition, the different locations of the hydrophobic patches in the bracelet cyclotides and Möbius cyclotides determine their different binding orientations [84]. Studies on cyclotide–membrane interactions revealed that cyclotides have a high specificity for PE-containing phospholipids over other lipids, and insert into the hydrophobic core of the membrane rather than just adsorb at the phospholipid headgroup region. This result supports the notion that antiviral activity of cyclotides is probably mediated by disrupting the membranes in HIV-infected cells by pore formation, also correlated with the observed cytotoxicity [81, 85, 86]. PE is also present at the surface of enveloped viruses because viruses acquire their lipid membrane of the cells from which they bud off. It was reported that kalata B1 has virucidal activity, i.e., it can directly destroy the viral particle by disrupting the membrane envelope, a raft-like membrane with a high proportion of PE-containing phospholipids [80]. So, it is still unclear whether cyclotides exert antiviral activity through a mode of action that involves binding to the infected cells membrane, to the viral envelope, or to both [87]. In addition, the bracelet cyclotide tricyclon A does not possess the typical hydrophobic patch found in other cyclotides [70]. As mentioned above, the hydrophobic patch is important for their membrane-binding orientations, so whether it has a different binding mode needs to be explored. Whether cycloviolacin VY1, the only cyclotide discovered with anti-influenza A H1N1 virus activity, has a different antiviral mechanism than other anti-HIV cyclotides is still an open question. In summary, phospholipids seem to be the main antiviral target of most cyclotides, but different cyclotides may have different features in their mode of action. Their small size facilitates recombinant expression and chemical synthesis [87], which provides a basis for fully revealing the binding specificity and exact mechanism of a broad selection of cyclotides.

### Other antiviral RiPPs

Until now, there have been 38 classes of RiPPs discovered [19]. In addition to the classes discussed above, there are several single examples with antiviral activity reported of other RiPPs classes in recent years.

Proteusins represent a new RiPPs family composed of largely genome-predicted bacterial peptides carrying numerous unusual post-translational modifications. For a long time, the only known proteusins were the polytheonamides. But recently, a novel proteusin, landornamide A, was heterologously expressed and characterized. Landornamide A contains lanthionines, D-residues, and, unusually, two ornithines converted from arginine (Fig. 2). This latter conversion was regarded as a new post-translational modification (PTM) in RiPPs. What is even more remarkable is that landornamide A shows inhibitory activity against Lymphocytic choriomeningitis virus (LCMV) infection in mouse cells at low concentrations without toxicity towards the host cell [88]. The further structure–activity relationship study shows that rather than ornithines, cyclic lanthionines are essential for LCMV inhibition activity and that residues 8 and 15 are involved in their inhibitory function [88]. LCMV belongs to the family of arenaviruses, which cause various diseases in humans, such as meningitis, encephalitis, and abortion [89]. Ribavirin was the only reported anti-arenaviral compound before [90]; thus, the discovery of Landornamide A is of great significance. In addition, this work also provides a good starting point for exploring new PTMs and pharmaceutical bioactivity in abundant uncharacterized genome-predicted proteusins.

Thiopeptides are RiPPs that typically feature a 6-membered nitrogenous ring. This central ring serves as a scaffold for at least one macrocycle and a tail, and both can be decorated with various dehydroamino acids and azoles [91]. Thiopeptides usually display nanomolar concentration growth suppression activity towards Gram-positive pathogens by blocking protein translation [92]. But they are far from limited to their antibacterial activity, as a recent study found that one thiopeptides member, micrococcin P1, possesses inhibitory activity against Hepatitis C virus (HCV). It can efficiently inhibit HCV entry in a pan-genotypic manner with an EC_50_ range of 0.1–0.5 μM, even inhibiting sofosbuvir-resistant HCV strains [93]. The antiviral mechanism of micrococcin P1 was revealed by selection for viral drug resistance. The result showed it inhibits HCV entry at an attachment step by interfering with glycoprotein E2, the putative target. Furthermore, the authors verified that micrococcin P1 can also efficiently block cell-to-cell transmission [93]. Thus, micrococcin P1 interferes with HCV spread by efficiently blocking both direct infection and cell-to-cell transmission. As currently approved direct-acting antivirals against HCV exclusively target HCV RNA replication, micrococcin P1 with its novel antiviral mechanism offers promising potential to be developed as an anti-HCV drug to prevent the infection and spread of the HCV in the liver. In addition, the specific mechanism of micrococcin P1 allows it to potentially be used together with other antivirals in HCV combination therapies to improve treatment efficiency, as will be discussed in the next section.

Sactipeptides are RiPPs typified by thioether linkages between a cysteine-sulfur and the α-carbon of another residue (Fig. 2). It represents a small but growing class with six peptides characterized. While all these peptides show some antimicrobial activity, subtilosin is the only one found to have antiviral activity. Subtilosin exhibits efficient anti-HSV activity in a broad concentration range [94, 95]. A virucidal assay verified that subtilosin has a direct virucidal effect on HSV-1 and HSV-2 particles at high concentration (200 µg/mL), but the inhibition effect at low concentration (≤ 100 µg/mL) does not come from a direct effect on the virus. This suggests that subtilosin uses a different mechanism of action at different concentrations to inactivate or inhibit the virus. Initial characterization of the inhibitory action of subtilosin at low concentration showed that subtilosin does not interfere with early stages of viral multiplication [94]. Subsequently, the mechanism was further revealed. Subtilosin may block late stages on the viral replicative cycle, since it impedes not only virus release but also viral particle formation. While the exact antiviral target is still uncertain, the viral glycoprotein gD is the possible target [95]. Commonly used drugs to treat herpes infections, such as acyclovir and penciclovir, selectively inhibit the viral DNA polymerase activity. Subtilosin, with its different mechanism of action, has been shown to inhibit the multiplication of acyclovir-resistant strains of HSV-1 [94], raising the potential of using subtilosin in HSV treatment.

Cyanobactins are a large class of RiPPs produced by cyanobacteria. They have traditionally been described as cyclic N-to-C macrocyclized peptides containing heterocyclized amino acids. However, examples of short, linear cyanobactins have also emerged [96]. Cyanobacterial compounds are often associated with their biological activities, such as cytotoxicity, antimalarial and antiviral activities [97]. Dolastatin 3 is a cyanobactin which was found to have anti-HIV activity as part of screening new inhibitors of HIV-1 integrase from marine organisms [98, 99]. In this work, dolastatin 3 crude extracts were reported to inhibit HIV integrase. However, due to multiple experimental differences and its cytotoxicity, no further antiviral activity studies of dolastatin 3 were conducted [99].

The natural-occurring antiviral RiPPs from different RiPPs classes have different structural and activity properties, determining their different mechanism of action (Fig. 1) and, therefore, show different advantages as antiviral drugs. For instance, duramycin and labyrinthopeptins from the lanthipeptide class can be developed as broad-spectrum antiviral drugs because of their PE-targeting mode of action. The antiviral class I lasso peptides have remarkable stability and show a high degree of structural similarity. This structural rigidity makes them highly promising leads or templates for the design of RiPPs-based anti-HIV agents. The antiviral cyclotides possess useful biopharmaceutical characteristics including exceptional stability and membrane permeating activity. The high tolerance to sequence variability of cyclotides enables them to be used as a novel framework for antiviral drug design and drug delivery. Thus, RiPPs have great potential in antiviral prevention and treatment. It is worthwhile to deeply study the structure–activity relationship and mechanism of action of these antiviral RiPPs. In addition, drug delivery and treatment methods and further safety assessment are needed to develop them as pharmaceutical agents.

### Increasing the potency of RiPPs as antiviral agents

The resistance to proteases, high specificity and affinity for targets, and membrane permeability make RiPPs good candidates for novel therapeutic drugs. However, in many cases, there is still room for improvement in bioavailability and safety of antiviral RiPPs. Several studies on optimizing RiPPs by various engineering strategies, or combining RiPPs with other antiviral drugs and/or nanobiotechnology, to improve their antiviral potency have been reported.

### Engineering of antiviral RiPPs

For most of the RiPPs with antiviral activity described in the previous section, their identification is dependent on natural product discovery efforts, which mainly employ two approaches. First is a ‘top-down’ approach driven by the screening of biological samples for desirable bioactivities, followed by compound isolation and characterization [100]. Another genome-driven discovery approach was developed based on genome sequencing technologies, by analyzing the genomes of natural organisms through bioinformatics methods to evaluate their biosynthetic potential and identify clusters of new natural products [22, 101]. However, these natural discovery efforts rely partially on luck, and many technical challenges set up roadblocks for the efficient discovery of novel RiPPs with expected pharmaceutical potential.

The shortcomings of these natural discovery approaches can be overcome by RiPPs engineering. Recent advances in bioengineering and molecular biology make it possible to manipulate the synthesis pathway of precursor peptides and biological synthesis of RiPPs using heterologous expression systems, enabling the generation of large genetically encoded libraries of RiPPs for selecting expected properties in a high-throughput way [20, 21, 102–105]. In addition, RiPPs biosynthetic pathways are modular and intrinsically tolerant to alternative substrates [106, 107]. This biosynthesis plasticity allows for various efforts on RiPPs bioengineering, including changing the amino acid composition, charge, topology, and other chemical and structural characteristics that may influence the bioactivity of RiPPs. Moreover, different chemical engineering strategies are also being developed to synthesize and modify RiPPs [19]. RiPPs engineering facilitated the study on structure–activity relationships, and more importantly these in vivo and in vitro engineering systems created RiPP analogs with altered or enhanced antiviral activity, antiviral spectrum and pharmaceutical properties (e.g., higher bioavailability and lower hemolytic activity), such that they are more suitable for utilization in antiviral therapy [29, 108–111].

**Fig. 3:**
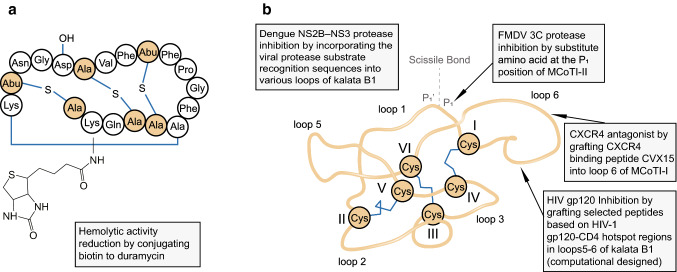
Examples of some of the engineered modifications made to RiPPs to optimize or create novel antiviral activities. **a** Biotinylated duramycin; **b** Modifications made to the kalata B1, MCoTI-I and MCoTI-II framework. The locations of the modifications introduced into the cyclotide framework are illustrated using the MCoTI-II structure (pdb ID: 1IB9)

Cyclotides are the most studied antiviral RiPPs in engineering research. Although most of the current engineering efforts are aimed at exploring structure–activity relationships, the use of the cyclotide scaffold in antiviral drug design, either by changing their specificity of inhibition or creating new binding activity, has also been shown recently. The first example is the engineering of a range of substitutions at the P_1_ position of trypsin inhibitors MCoTI-I and MCoTI-II, which is depicted in Fig. 3b [110], producing MCoTI-II analogs with redirected specificity towards alternative protease targets. Remarkably, several MCoTI-II analogs exerted selective low-μM range inhibition of 3C protease from Foot to mouth-disease virus (FMDV 3Cpro), an identified cysteine protease essential for replication of this virulent pathogen. Another example of engineering of cyclotides for changing the specificity of inhibition is provided by the Möbius cyclotide kalata B1 [108]. The NS2B–NS3 protease plays important roles in post-translational proteolytic processing of the Dengue viral polyprotein and in viral replication and maturation, and it is an attractive target for anti-Dengue viral drug design. For discovering potent Dengue NS2B–NS3 protease inhibitors, the anti-HIV Möbius cyclotide kalata B1 was engineered to generate analogs by varying the amino acid sequence (Fig. 3b). Various analogs were evaluated for their inhibitory activities against Dengue NS2B–NS3 protease. One engineered kalata B1 that exists in two distinct oxidized forms showed potent inhibition, making it a promising candidate to be developed as NS2B–NS3 protease inhibitor. The substitution of amino acid residues in variable loop regions provides kalata B1 with protease inhibitory activity in this example, while, the membrane-disruptive activity of native cyclotides is mainly dominated by their CCK motif. Although the membrane-binding and disrupting ability of these kalata B1 anologs have not yet been assessed and compared, it provides the possibility for producing novel cyclotide-based antivirals with a dual mode of action, i.e., membrane-mediated action and protease inhibition action.

Phospholipids have been reported to be the main antiviral binding target of most natural-occurring cyclotides. However, engineered cyclotides bearing a new binding activity and enhanced antiviral activity by epitope grafting have also been also reported. For example, chimeric MCoTI-Is are able to efficiently inhibit HIV-1 viral infection by selectively targeting cytokine receptor CXCR4, a co-receptor for viral entry into host cells [109]. In this study, different sequences of a CXCR4 binding peptide CVX15 were grafted into loop 6 of MCoTI-I (Fig. 3b). All engineered MCoTI-Is were able to block CXCR4 activation in a dose-dependent manner, and the most active compound (MCo-CVX-5c) among them was regarded as a potent CXCR4 antagonist with EC_50_ around 20 nM. MCo-CVX-5c also inhibited the replication of HIV-1 with EC_50_ around 2 nM, in contrast to naturally occurring MCoTI-I that did not show any inhibitory activity. In another study, anti-HIV epitopes were grafted onto cyclotide kalata B1, in an effort to reduce its toxicity and simultaneously improve its anti-HIV activity [111]. Loops 5 and 6 play an important role in the antiviral binding and hemolytic activity of kalata B1, thus genetic algorithms were used to select peptides based on HIV-1 gp120-CD4 hotspot regions for grafting in between these two loops which was followed by molecular modeling techniques to assess the chimeric kalata B1 (Fig. 3b), finally screening out two modified kalata B1s that exhibited better interaction energies (36.6% and 22.8%, respectively) when binding to HIV-1 gp120 compared to wild-type kalata B1, in spite of no in vitro validation. These results open the way for engineering cyclotides-based antivirals with different binding activity and enhanced inhibitory activity.

Some antiviral RiPPs such as duramycin cause cytotoxic effects and significant hemolysis, dampening their therapeutic potential. Thus, reducing toxicity is also an important goal in RiPPs-based drug design. There is an example of modifying duramycin by conjugation with biotin, aiming for it to be used as indicator to determine the distribution of PE on endothelial cells. Surprisingly, the biotinylated duramycin did not change its affinity nor specificity of binding to PE, but reduced the hemolytic activity by fourfold compared to duramycin (Fig. 3a) [29]. The water-soluble biotin moiety increased the hydrophilic character of peptide was suggested to be possible cause of this result [29]. This finding provided a rational way for engineering duramycin to reduce its hemolytic activity, thus improving the therapeutic index for potential clinical use.

There is a growing number of engineered RiPPs that show enhanced or new biological activity, which makes them more attractive from a pharmaceutical perspective. Work from our lab illustrates various examples of employing RiPP biosynthetic machinery to engineer nisin-based lantibiotics with increased antimicrobial activity, and producing novel RiPPs with antimicrobial activity [21, 107, 112–114]. With increasing interest to the antiviral activity of RiPPs, these works provide a platform/reference for engineering RiPPs to improve their antiviral activity. Recently, an article described the production of a genetically encoded library of 10^6^ lanthipeptides in *Escherichia coli* using the RiPP biosynthetic enzyme ProcM [115]. This lanthipeptide library was coupled to a bacterial reverse two-hybrid system for screening inhibitors of the interaction between HIV p6 protein and the UEV (ubiquitin E2 variant) domain of the human TSG101 protein. This interaction is critical for HIV budding from infected cells, and is a potential target for antiviral therapy [116]*.* An inhibitor was identified from the lanthipeptide library, the activity of which was verified in vitro and in cell-based virus-like particle-budding assays. This study, utilizing a RiPP biosynthetic machinery, provides a good approach for identifying novel RiPPs with antiviral activity and genetically engineering RiPPs with improved antiviral activity.

Given the high antiviral potential, remarkable stability and excellent tolerance to sequence variations of naturally occurring antiviral RiPPs, there are still many ongoing bioengineering and chemical modification efforts on these RiPPs to generate novel antiviral drugs. For instance, a patent publication states that a lot of labyrinthopeptin derivatives can be used for both treating existing viral infection and for preventing the occurrence of a viral infection. Said viral infection may be an infection with any one of the viruses selected from Respiratory Syncytial virus, Kaposi Sarcoma-associated Herpes virus, Cytomegalovirus, Dengue virus, Chikungunya virus, Tick-borne Encephalitis virus, Vesicular Stomatitis Indiana virus, Zika virus and HCV [117]. All above results indicate that engineering of RiPPs can successfully be used for the development of new RiPPs-based antiviral drugs.

### Combination therapy with other antiviral agents

To improve the effectiveness of treatment and prevent resistance, combination regimens have been given increased attention in clinical development. RiPPs can be developed as either direct-acting antivirals or as indirect inhibitors combined with other antivirals, to avoid to a certain extent the high dose requirements, the development of drug resistance and the limits of antiviral efficacy when RiPPs or other drugs are used alone. A series of strategies has been developed for RiPPs combination therapy to improve its prospects in antiviral therapy.

Improving the efficacy of drugs is an important goal in combination therapy. Labyrinthopeptins act as entry inhibitors against various viruses. It has been demonstrated that LabyA1, in combination with clinically approved drugs such as enfuvirtide (fusion inhibitor), raltegravir (integrase inhibitor) or tenofovir (reverse transcriptase inhibitor), result in a synergetic effect. Also, in combination with the experimental gp120-targeting peptide griffithsin, LabyA1 showed moderate synergy [43]. Another RiPP, micrococcin P1, which inhibits HCV entry at an attachment step [93], showed synergistic antiviral effects in combination with other clinically available HCV drugs (targeting HCV RNA replication) [93]. The RiPP cycloviolacin O2, with ability to disrupt the integrity of viral particles, was also verified to increase the efficacy of the entry inhibitor drug enfuvirtide [79]. Taken together, because of their particular mode of action, the combination of RiPPs with standard drugs, ideally targeting complementary steps of the viral life cycle, could be developed as a strategy to improve therapeutic efficacy. Rapid development of resistance is a big challenge associated with nelfinavir and other helicase-primase inhibitors (HPIs) for use in HIV treatment. The combination of cycloviolacin O2 and nelfinavir have an additive effect on reducing viral load, verifying that cycloviolacin O2 may be used as an adjuvant to enhance the efficacy of HPIs in HIV-infected cells [71]. Thus, the combination of RiPPs with commonly prescribed antiviral agents provides an efficient strategy to prevent resistance development. Moreover, it has been shown that combined use of cycloviolacin O2 with saquinavir was able to reduce the dosage required of the latter drug in inhibiting HIV-1 replication [79]. At low concentrations, saquinavir or cycloviolacin O2 alone did not significantly inhibit HIV-1 replication; however, co-exposure of them enabled a remarkable suppression in HIV-1 p24 levels, and almost a total suppression of HIV-1 p24 was seen following exposure to this combination for 6 h.

Studies on cell-penetrating peptides (CPPs) revealed that these short peptides can be successfully utilized to enhance the cellular uptake and intracellular trafficking of antiviral molecules. Considering that some antiviral RiPPs such as lanthipeptides and cyclotides have good cell-penetrating activity, they appear to be good candidates to increase the inhibitory activity of other direct-acting antivirals in a similar fashion to CPPs. The cyclotide cycloviolacin O2 can significantly increase the intracellular uptake of saquinavir in cycloviolacin O2 pre-treated cells. The combined use of nisin and lactoferrin resulted in a strong anti-BVDV effect on both the extracellular viral titer and the intracellular viral RNA level [23]. Although there is no experiment to prove that nisin acts as CPP in this combination to improve the antiviral efficacy of lactoferrin, it occurs potentially through this mechanism. These results suggest that RiPPs can be developed as CPPs to play a role in antiviral combination therapy to help cellular entry of other antiviral agents, especially large molecular weight drugs.

### Combination with drug delivery technologies

Although the combination of multiple drugs in active therapy has significantly improved antiviral efficiency, the constant threat of emerging drug resistance and the limitation of drug bioavailability are still pushing the development of new antiviral strategies. Nanotechnology-based drug systems for antiviral treatment represent an important option [118]. Nanomaterials display remarkable physical and chemical properties, e.g., the nanometric size (beneficial for drug delivery through impermeable barriers), large surface area to volume ratio (beneficial for large drug loads and enhanced solubility), and their tunable surface charge (for drug encapsulation). Additionally, combined use of nanomaterials and antiviral agents may circumvent drug resistance mechanisms [116, 117]. Some nanomaterials including nanoparticles, liposomes, nanospheres and nanogels have been studied either in vitro or in vivo for drug delivery of antiviral agents, and recent reviews present an overview of antiviral nanotherapeutics [118, 120]. The interaction between nanomaterials and antiviral RiPPs is an interesting field to be investigated and developed. An example of the use of nanotechnology in combination with antiviral RiPPs is subtilosin-based nanofiber. Subtilosin has anti-HSV-1 activity and it can be readily encapsulated into Poly-vinyl alcohol (PVOH) nanofiber mats, a water soluble and non-toxic biocompatible polymer. The high viability (98.5%) for the human skin tissues exposed to subtilosin-based nanofibers illustrates the safety of this formulation and its potential for use in human applications [94]. However, further study is needed to assess the anti-HSV-1 activity of subtilosin-based nanofibers.

An example of applying nanomaterials to improve the bioavailability of RiPPs is improving the oral availability of labyrinthopeptin. The hydrophobic characteristic of labyrinthopeptin makes that it has poor solubility and bad oral availability. However, labyrinthopeptin can be solubilized using nanoparticles called GCPQ (Quaternary ammonium palmitoyl glycol chitosan) and oral uptake of labyrinthopeptin was improved in vivo by this formulation. Moreover, the characteristics of GCPQ can be modified to control the uptake of labyrinthopeptin further [121]. Although the treatment effect evaluated in this study was directed to anti-nociceptive activity, it also provided a reference formulation for the improvement of antiviral treatment effect of labyrinthopeptin. In summary, nanoparticles associated with antiviral drugs allow enhanced bioavailability and safety, and even controlled release kinetics. Nanomedicine formulations provide an alternative for the development of antiviral RiPPs in the future. Other technologies and materials are also being developed for new antiviral formulas, such as tenofovir in a gel formulation, which appears safe and effective in preventing HIV infection, and has great potential as a antiretroviral microbicide [122].

### Screening methods to identify novel antiviral RiPPs

As a superfamily with numerous members, RiPPs form an important source for drug discovery, but research on antiviral activity of RiPPs is still rare, although there is great research potential for RiPPs with antiviral activity. In this part, we will discuss the screening methods and design principles that could be used in discovering novel antiviral RiPPs.

Computer-aided structure-based drug discovery has become a very important part of the rational drug screening/design process nowadays, which involves the advancement of pharmacophore modeling methods and structural biology techniques [123]. Pharmacophore modeling can be established either by structure-based modeling, or by ligand-based modeling. Structure-based modeling uses available three-dimensional (3D) structures of a target, which are provided by X-ray diffraction, nuclear magnetic resonance (NMR) or molecular simulation, and adopts a series of methods, such as molecular docking to analyze physicochemical properties of a drug binding site on target and evaluate binding interactions [124–127]. This is followed by screening of a small molecules library to provide possible drug candidates [123]. Ligand-based modeling, on the other hand, only relies on active ligand information for analyzing the structural requirements of the ligand and optimizing the interaction, followed by designing novel analogs of known active ligands and predict activity [128, 126]. A recent study using computer-aided tools predicted several antiviral peptides derived from the antiviral RiPPs nisin and subtilosin [74], providing an example of the use of molecular docking to test the affinity between the predicted peptides and the HEV capsid protein and to establish the best interactions between them. Eventually, a potential antiviral agent derived from subtilosin was screened out. Another example combined molecular modeling techniques with a genetic algorithm to automate the design of new cyclotides with improved binding to HIV gp120 [111]. Various different strategies have been developed to manipulate RiPP biosynthetic pathways, which makes it possible to successfully synthesize RiPPs-based antivirals [19, 102, 107]. These new peptides could be evaluated in vitro*,* subsequently. In general, computer-aided structure-based drug discovery increases the hit rate and reduces the time and cost of drug development, making it one of the more effective means to discover lead antiviral RiPPs.

High-throughput screening is a more direct method to isolate desired molecules, compared to virtual computer-aided discovery methods, accelerating the process of novel antiviral drug discovery. High throughput screening assays fall into two major categories, i.e., biochemical assays and cell-based assays. Biochemical assays are based on purified viral targets, and often directly and specifically measure the affinity of the test compound for the target of interest [130]. In contrast, cell-based assays usually interrogate an intracellular viral target to identify small molecular inhibitors and evaluate the cytotoxicity of these molecules. While the screened molecules could quickly prove to be useful in drug therapy, to elucidate the specific mechanism of action requires follow-up research [131]. For example, as mentioned in the previous section, a cell-based assay was used to screen a genetically encoded library of 10^6^ lanthipeptides in *Escherichia coli,* to identify inhibitors of interaction between HIV p6 protein and UEV domain of the human TSG101 protein, a potential target for antiviral therapy. Subsequently, the inhibitory activity of the identified inhibitor was further verified in vitro [115]. Such combination of RiPPs libraries with different cell-based assays could be developed to identify a larger number of antiviral RiPPs. Overall, the ongoing efforts on RiPPs screening and engineering modifications hold great promise in identifying novel antiviral RiPPs that can be used, alone or in combination, for activity against various steps in the virus life cycle.

### Suitability of RiPPs as antiviral therapeutics

It should be noted that the antiviral application of RiPPs appears to be still in a pioneering stage. As a result, in vivo data on effects in the human body are largely lacking in the studies reviewed here. Activity and toxicity tests were often performed using in vitro cell cultures. Nevertheless, the suitability of therapeutic use of RiPPs as antibacterial agents as well as unmodified peptides as antibacterial and antiviral agents in humans has been investigated before. Certain findings concerning the therapeutic use of modified and unmodified peptides are general, regardless of their target. They can, thus, be applied to explore RiPPs as antivirals as well. By relating these points to specific details of the antiviral RiPPs covered here, we will provide a prospect on future clinical use.

### Effects on the microbiota of the body

Because RiPPs are associated predominantly with antibacterial activity, the possibility of secondary effects on the beneficial microbiota of the body comes to mind. Ideally, the target specificity of a RiPP that is to be used as antiviral agent should be specific enough to not target the commensal bacteria. These concerns were discussed for the use of unmodified peptides as antibacterial agents, and it was noted that the restricted specificity of said peptides reduces this risk [132]. It can be imagined that the selection of candidate RiPPs purely based on their antiviral activity is already a step in the right direction. However, some RiPPs may have a dual activity. For example, the cyclotide cycloviolacin O2 also has potent antibacterial activity [71], as does the lanthipeptide nisin [23]. On the other hand, there are examples of RiPPs with antiviral activity that lack activity towards relevant commensal strains of bacteria. In one study, several vaginal commensal *Lactobacillus* strains and a gastrointestinal strain, *L. rhamnosus* GG, were subjected to the anti-HIV/anti-HSV lanthipeptide labyrinthopeptin A1. Growth inhibition was not observed, at concentrations up to 120 µM. Nisin, on the other hand, killed many of the strains at lower concentrations [43], although this could be overcome by using inactive nisin fragments or otherwise modified nisin species that still would retain antiviral activity. This illustrates that adverse effects toward the microbiota may not always have to be present for antiviral RiPPs. However, in cases where the possibility of dual activity of a RiPP is inevitable, the effects on the microbiota can still be minimized in different ways. With respect to the therapeutic application of lanthipeptides, it is noted that their low absorption rates enable local delivery in the body. An advantage of this is that the effect on the microbiota elsewhere in the body may be reduced this way [133]. Of course, it should be investigated whether this is true for RiPPs in general, including those covered here.

### Suitability of treatment route

The delivery route of antiviral RiPPs is not only important for the effect on microbiota, but also crucial for effectively inhibiting viral infection. Various ways of locally administering peptides to the body have been developed, such as oral administration through tablets, dermic application, inhalation, infusion or intravaginal application [133]. Even though these examples may not be specifically developed for antiviral RiPPs yet, it is likely that they will be applicable for antiviral RiPPs too in the future, because of their similar peptidic nature. Still, the best treatment route truly depends on the specific virus to be targeted. It should of course be relevant to the location where infection of a particular virus generally takes place, but also to the stage of the infection pathway at which the RiPP is active. For example, if an antiviral RiPP is active by lysis of virions, or inhibiting attachment to the host cells, inhalation may be best suited for the prevention or treatment of early stage respiratory diseases. In general, systemic application of therapeutic peptides is faced with a few hurdles. Properties like molecular size, solubility and stability can play a role in deciding which administration methods are best suited for RiPPs application. For unmodified peptides, rapid degradation presents a major problem [13]. However, this is less of an issue for RiPPs, as they have superior stability. The cysteine knot in cyclotides provides an example of a very stable motif that grants resistance to proteolysis [61]. Kalata B1, and engineered cyclotides based on Kalata B1, have been proven to be orally bioactive [87]. Similarly, the class I lasso peptide RP71955 is highly resistant to hydrolysis. As a result, it is a good candidate to be administered through oral absorption [50]. In contrast, thiopeptides, like micrococcin P1, seem limited to topical treatment, due to their large size and poor solubility in water [91]. Some lanthipeptides bind plasma proteins and blood cells, which is a challenge for their bioavailability [133]. This effect should also be studied for the other classes of antiviral RiPPs, as it can affect the dosage of these peptides too. To improve delivery of antiviral peptides, conjugation with antibodies, nanocarriers, carbohydrates or lipids has been suggested [13].

Several specific applications are proposed for antiviral RiPPs, enabling a more targeted and localized effect. For the anti-HCV RiPP micrococcin P1, a role is suggested in prevention of viral spread and reinfection during liver transplantation [93]. However, their limited uptake, as discussed, should be kept in mind. In one study, intrapulmonary administration of nebulized duramycin was achieved in an effort to evaluate its potential use in the treatment of cystic fibrosis [134]. As mentioned in a previous section, combinatorial use with nanomaterial greatly improves the solubility of labyrinthopeptin as an oral formulation [118], and enables subtilosin to be applied directly to areas infected with HSV-1 [94]. What could be interesting is a probiotic approach for the administration of antiviral agents. For example, a *Lactobacillus* strain expressing labyrinthopeptin A1 is put forward to provide anti-HIV and anti-HSV activity [43]. Similarly, the probiotic strain *Enterococcus faecium* L3, which produces enterocin B, provides a protective effect against influenza infection in mice, possibly through effects on the immune system and microbiota [135]. Indeed, bacterial in situ production may be a good strategy to ensure sufficient bioavailability of antiviral agents, at the location where it is needed. Probiotic production or technologically advanced delivery methods are on the rise and could provide innovative ways to deliver RiPPs at the right place in the near future. Mostly, there is some flexibility in the molecular properties of RiPPs, and they can be tweaked to reduce the limiting and enhance the beneficial characteristics.

### Toxicity

Apart from negative side effects on the microbiota, the possibility of toxic effects on the human body should also be considered. This concern is addressed in the reviewed studies on antiviral RiPPs, for instance by assessing hemolytic or cytotoxic activity [40, 63, 68, 74, 88]. In general, it seems that the effective concentration of the drug is often well below the toxic concentration. Low cytotoxicity is for instance reported for RP71955 [50]. Humidimycin reportedly lacks cytotoxicity in vitro [58], as is true also for landornamide A [88]. And as mentioned before, the Möbius subfamily of cyclotides, of which Kalata B1 is a good example, has anti-HIV activity that is comparable to the bracelet subfamily, but with lower cytotoxicity [74].

Still, the toxicity of antiviral RiPPs should be individually assessed, assuring that the concentration range that separates the antiviral effects from the cytotoxic activity is wide enough, as indicated by the therapeutic index [61]. Several factors are beneficial to this therapeutic index of RiPPs. As noted, the dosage needed can be lowered through combination therapy [93]. Their composition allows breakdown eventually into non-toxic amino acids [132]. For certain cyclotides, it has been mentioned that variations in their sequence can affect cytotoxic activity while leaving the proteolytic stability unaffected [68]. Similarly, a study on divamides reported that simple modifications of the peptide sequence can have a large influence on the therapeutic index [40]. Thus, toxicity of antiviral RiPPs can be tweaked through engineering of the peptides, highlighting again the benefit of the gene-encoded nature and ease of obtaining variant molecules of RiPPs. The most important quality that determines toxicity may be the specificity of the agent. For example, the anti-HCV RiPP micrococcin P1 has antibiotic activity against prokaryotes and protozoa through inhibition of protein synthesis. However, as eukaryotic ribosomes are unaffected, the chances of side effects in humans seem small [93].

### Antiviral resistance

The likelihood of development of antiviral drug resistance to RiPPs is an important issue to be addressed. While data on viral drug resistance development to RiPPs are largely lacking, some general remarks can be made. Ideally, antiviral RiPPs should have a target that is crucial for the viral life cycle, so that the chance of circumventing this dependency through viral mutations is minimized. In this way, the low incidence of bacterial resistance to nisin can be explained, as it targets the conserved lipid II [136]. In a study on the anti-RSV RiPPs labyrinthopeptin A1 and A2, no cross-resistance by mutations that cause resistance to commonly used anti-RSV drugs was observed [44]. Similar results were found for micrococcin P1 [93] and subtilosin [94]. While these observations support the case for using these compounds in novel treatments, it does not reveal much about the likelihood of resistance development, as they do not test selection pressure for resistance against those drugs. It does, however, show that the targets of the novel drugs are different enough from those targeted by traditional treatments, so that cross-resistance is unlikely. It was found that prolonged exposure of HIV-1 RF to increasing doses of siamycin I selected for a resistant virus variant that showed a nine-fold decreased susceptibility to the drug. Sequencing revealed that the selection had resulted in the change of six amino acids in the gp120 and gp41 domains [57]. The question remains whether the resistance development observed is clinically relevant. The selection started with doses of siamycin I well above the ED_50_, took place over a period of 6 months, and apparently required multiple mutations. These conditions might be exaggerated compared to a clinical setting.

Ultimately, the chance of resistance development depends on the target, and this should be further investigated for each antiviral RiPP candidate separately. One of the main questions will then be whether the target is relatively conserved. With respect to the chance of resistance development, it can also be imagined that use in combination therapy would benefit the case of RiPPs. Attacking the virus at multiple targets in a single treatment would likely lower the chances of resistance arising through mutation of a single target.

## Conclusions

Vaccines and antiviral small molecules are usually preferred options for the prevention and treatment of viral infections. The enthusiasm for therapeutic use of peptides was tempered by certain weaknesses of linear peptides, including vulnerability to degradation, short half-life, and lack of selectivity. However, RiPPs commonly have cyclic structures conferred by post-translational modifications, which impart them with remarkable proteolytic stability, binding specificity, membrane permeability and even better oral availability. Especially some RiPPs that target essential components of the membrane that makes up the viral envelope are interesting leads for the development of antiviral RiPP therapeutics. Through targeting a conserved component of the membrane, resistance development may be slowed down, while a broad activity spectrum could be achieved at the same time. There are, of course, some challenges that cannot be ignored, such as segregation of the antiviral and cytotoxic properties of some membrane-active RiPPs; possibility of drug resistance triggered by continuous use, and the current lack of in vivo clinical tests. These challenges should be addressed by further exploration of RiPPs antiviral activity both in vivo and in vitro*,* to determine exact mechanisms of action and resistance development. There is no doubt that the high plasticity of RiPP biosynthesis could help expedite this process, allowing for production of potent novel antivirals. In conclusion, RiPPs may change the situation of peptide-based therapy and become important candidates for antiviral drug development, providing an innovative, highly promising, and cost-effective option for the treatment of viral disease.
